# Publication dynamics: what can be done to eliminate barriers to publishing full manuscripts by the postgraduate trainees of a low-middle income country?

**DOI:** 10.1186/s13104-022-06138-5

**Published:** 2022-07-15

**Authors:** Hafsa Majid, Lena Jafri, Sibtain Ahmed, Muhammad Abbas Abid, Mohammad Aamir, Aamir Ijaz, Aysha Habib Khan, Imran Siddiqui

**Affiliations:** 1 Section of Chemical Pathology, Department of Pathology and Laboratory Medicine, Aga Khan University, Stadium Road, P.O. Box 3500, Karachi, 74800 Pakistan; 2 Department of Pathology, Armed Forces Institute of Pathology, Rawalpindi, Pakistan; 3 Department of Pathology, Bahria International University, Rawalpindi, Pakistan

**Keywords:** Publication, Barriers, Scientific writing, Challenges, Manuscript, Low-middle income country

## Abstract

**Objectives:**

This study aimed to determine the publication rate of free paper abstracts presented by the postgraduate (PG) trainees and determine the reasons for non-publication. A mixed methods study was conducted. PG trainees presenting free papers at the at the Pakistan Society of Chemical Pathologist conferences from 2012 to 2018 were included. Three databases were searched to identify if the abstracts were published or not. The PG trainee authors of abstracts not published as full manuscript, were surveyed to determine the barriers and challenges in publishing a manuscript.

**Results:**

The average rate of full manuscript publication was 51.8% (n = 93/177) for the abstracts presented by the PG trainees. Publication rate was higher for oral (n = 73/119, 61.3%) compared to poster presentation (n = 20/58, 34.5%). Most of the manuscripts were published after two years of abstract presentation. The survey showed that the main challenges to publishing an abstract were lack of time, limited scientific writing or submission skills, lack of funding for publication fee, and negative or statistically non-significant results. This reflects a need to arrange workshops/symposia for the PG trainees of low-middle income country (LMIC) to enhance their writing and time management skills and improve the full manuscript publication rate from LMICs.

**Supplementary Information:**

The online version contains supplementary material available at 10.1186/s13104-022-06138-5.

## Introduction

Scientific meetings provide a platform for sharing developments in medical research, exchange ideas, and initiate future collaboration. Presentation of a study at a scientific conference is a method for rapidly disseminating research information which otherwise may take months or even years until it is made available to the colleagues through peer-reviewed journals [1]. Not all meeting abstracts eventually get published in a peer-reviewed journal. This is especially true for free paper abstracts presented by the postgraduate (PG) trainees of low-middle income country (LMIC), who have limited resources as well as experience in terms of writing and successfully publishing their research findings [2]. Previously reported publication rates in other medicine fields (percentage of abstracts presented in a meeting that were eventually published) range from 20.5% to 68.9% [3, 4].

In LMICs, where research funding is low and research opportunities are scarce, it is feared that the rate of publication could be even lower, especially in countries like Pakistan. This leads to a gap in the current literature from the LMICs and this will become more apparent seeing the 97% of population growth in developing versus the developed countries [5]. A study by Fazey I et al. reported that only one third of the literature is reported from lower income countries, and only half of these studies had primary authors from LMICs [6]. A survey by Journal of the Association of Nurses in AIDS Care reported that lack of time was the most common barrier, followed by lack of writing skills, lack of self-confidence, and a lack of motivation/ideas [7]. There is a need to inculcate research culture in PG trainees of LMICs and encouraging them to publish their research findings as full manuscript, as these trainees are the ones who will become future researchers and practitioners.

The annual conference of Pakistan Society of Chemical Pathologist (PSCP) is the largest national event fully dedicated to the analytical, clinical, epidemiological, and translational research areas of Chemical Pathology in Pakistan. Majority of submissions come from PGME trainees who are beginning their research career under supervision of senior faculty. In this study we aimed to determine the factors responsible for non-publication in our setup and what further steps can be taken to improve it.

### Methods

Mixed method study was conducted to ascertain the self-perceived barriers to full manuscript publication by the PG trainees. In the first phase, quantitative data of free paper abstracts by PG trainees accepted for presentation at the PSCP conferences from 2012 to 2018 was analyzed. To identify the maximum number of articles published, we choose to include abstracts from meetings done at least three years ago, as previous literature has reported that most articles are published as full manuscript within two to three years of presentation [3, 4]. Abstracts presented were identified from abstract books, previous conference program details, shared by Chair scientific committee or heads of institutes where conferences were conducted, or members of the previous conferences’ scientific committees.

A manual search of the online PubMed, Google scholar, and PakMedinet databases for each abstract using keywords (if not already defined, then selected from the body or title of abstract) and presenting author names of abstracts. If not found on the aforementioned search engines, authors were contacted to gather information about the full manuscript published. An abstract was considered published as a complete manuscript if the title, manuscript body, author list had similarities with the abstract presented. Journal titles, indexing, local or international (journal published from a country other than Pakistan), Medline/PubMed indexing status, impact factors and time to publication (months elapsed between the presentation at the conference and the publication of the manuscript) was retrieved for each published article.

#### Survey to identify barriers to full manuscript publication

For identifying the reasons of not publishing a 15-question anonymous survey was developed on Google forms and piloted to check the face validity, functionality and logic by two peers who were not part of the study (Additional file 1). The survey was shared with presenters via email, along with the title(s) of the abstract (s), year, and title of the conference. The survey was done from November 2020 to Jan 2021, including only authors of abstracts, whose full manuscripts were not found on the aforementioned search engines were contacted and periodic reminders were sent to improve the response rate.

#### Focus group discussion

A focus group discussion (FGD) with three young researchers with less than 5 years of experience after post-graduation, was conducted on March 13th, 2019 to determine the barriers and challenges in publishing abstracts presented at conferences, reasons of not publishing abstracts as full manuscripts and ascertain the possible solutions to these problems.

#### Ethical considerations

The study was done as per Helsinki’s ethical code, and the ethical review committee’s approval was not required. Exemption was sought from the institutional Ethical Review Committee (2019-1449-3769). A research assistant transferred all the research data to a password-protected computer that was not connected to the internet, via a USB flash drive. The computer was located in a research team member’s office.

#### Data analysis

Data was analyzed by Microsoft Excel 2010. Frequencies were generated for oral or poster abstracts, full manuscript published, local or international journal, high or low impact and reasons for non-publication. The publication rate for each conference was determined. A qualitative analysis of FGD data was performed.

## Main text

### Results

In PSCP meetings held annually over a 6 years’ period, a total of 177 free paper abstracts were presented. Of the total published abstracts 65.7% (n = 119) were presented as oral presentations. Of the total abstracts 51.8% (n = 93) of these were eventually published in journals. The overall publication rates for the 6 years ranged from 31.2% in 2017 to 77.8% in 2015 and the average time to publish was 1.71 years (Table 1).

**Table 1:** The overall publication rate for the annual PAP and PSCP

Year	Abstracts presented	Published	Publication rate (%)	Average time to publish after the conference (years)
2012	37	18	48.6	2
2013	12	6	50	1.8
2015	45	35	77.78	2.5
2016	13	8	61.5	1
2017	48	15	31.2	1.8
2018	22	11	42.3	1.18
Total	177	93	51.8	1.71

The publication rates for oral presentations were higher than poster presentations (61.3% vs 34.5%, p value 0.002) shown in Additional file 2: Fig. 1a, b. The publication rate in international journals was higher for abstracts presented as posters compared to oral 23.18% vs 11.48% and indexed journals publication rate was higher for poster abstracts as well 35.3% vs 29.32%.

#### Barriers to full manuscript publication

**Fig. 1:**
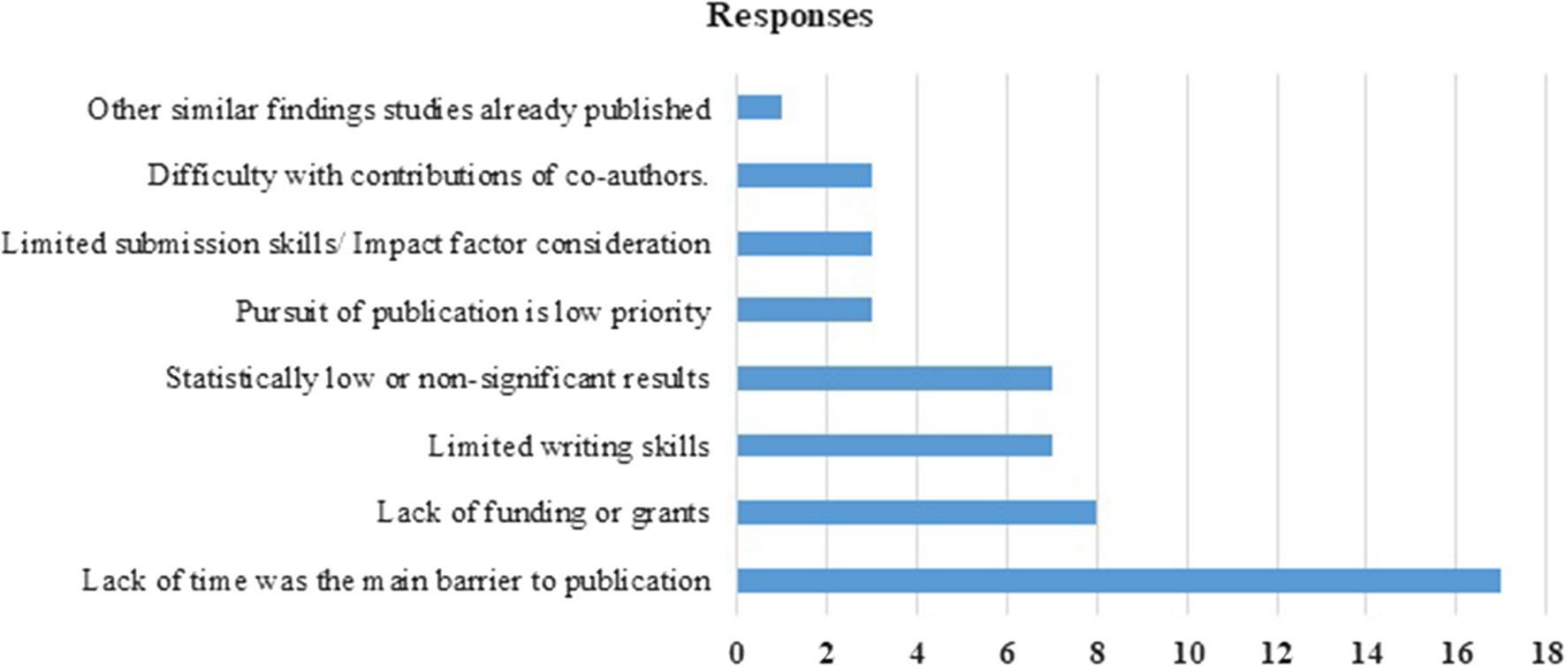
Self-reported barriers to publishing full text articles

The survey questionnaire was sent only to PG trainee authors, whose full manuscripts were not published as full manuscripts. Total 82 abstracts by 66 presenters were not published, and of these contact information was available for 60 presenters only. The response rate for the survey was 45% (27/60). The commonest barrier to publish an abstract as a full manuscript reported by the abstract authors was lack of time. The self-reported barriers to publication are shown in Fig. 1.

#### *Brainstorming *via* Focus Group Discussion*

A FGD was conducted to assess the challenges faced by the researchers while publishing researches. It was attended by the PI and three junior consultants from different medical universities across Pakistan. The consultants from AKU and Ziauddin University, Karachi participated in person, while residents from Quaid-e-Azam University, Bahawalpur, attended via Zoom. All the participants were presented with 7 open ended questions; and their responses are summarized in Table 2.

**Table 2:** Focus Group Discussion with three researchers to identify the barriers to publication

	1	2	3
When do you start writing manuscript?	After 2–3 months of paper presentation	After presenting it in a conference	• Before presenting it in a conference • After submitting dissertation • On supervisor’s inquiry
Time of manuscript writing	Weekends	Weekends, especially at night	no specific time of the day but on days when there is some free time
Challenges faced while getting manuscript reviewed by Co-authors	• Time taken (3–6 months) • Do not review properly • Conflict on Authorship Solution: • Finalize authorships on the day proposals are discussed • Work with likeminded people	• Time taken (2 to 3 months) • Co-authors assign task to residents • Lack of coordination Solution: The PI asks co-author to nominate 1 resident for liaison and coordination	• Time taken Solution: First send abstract to all co-authors. Clinicians only review parts which are more relevant to their specialty
How do you select the journal	• Prior affiliation of authors with journal • Article processing or Publication charges • Indexed/impact factor	• Journal aims and scope • Topic of research • Mostly local journal	• Time taken to publication • Journal’s publishing guidelines • Prefer international journal
Key factors leading to non-publication	• Time management • Not a requirement for exam, so lower on priority list	• Time management • Problems in getting ethical approvals, especially for retrospective studies	• Lower on list of priority • Problems faced in data analysis • Problems in getting ethical approvals
Why do you publish?	• For promotions	•For promotions •To disseminate research findings	• For promotion • To disseminate research findings
Suggestion to improve publication rate	• An indexed journal by PSCP • Increase local platforms/journal	• Encourage young researchers publish • Start working on manuscript after conference	• Need to identify the factors which prevent for non-publication • Working with senior faculty • Overcoming the mental obstacles

### Discussion

Publication of a free paper abstract as full text manuscripts not only improves discoverability of the work, widens dissemination, but the published objective evidence can then be used for developing guidelines and clinical practice recommendations. Only 44% abstracts presented were published. Similar publication rates were reported by other studies [8, 9]. Conference for the year 2014 was cancelled due to the unfortunate terrorist incident in a school in Peshawar.

Most common barrier to publication is lack of time. Kapp et al. reported that in addition to difficulties of the writing process, lack of time and uncertainty about choice of topic, the fear of rejection are also a major barrier to publication [10]. Keen et al. identified four questions that potentially challenge an aspiring writer (i) can I write already? (ii) what should I write about? (iii) who is going to read? and (iv) how should I write it? They state that the first question relates to personal confidence [11, 12]. The second question pertains to difficulties in development of ideas. The third question relates to the target audience and the selection of suitable journal for publication [13]. The fourth question reflects the uncertainties of the writing process itself and encapsulated numerous other barriers of the writing process [11]. Weathers et al. reported that the main barriers to publication for postgraduate year one pharmacy residents was lack of time, lack of knowledge of the publication process, lack of research mentorship, sub-standard quality of the study that is not suitable for publication, and delay in obtaining approval from the respective institutional review board [14]. All these studies reported barriers faced by academicians in different fields. Even though we report challenges faced by presenters at the Pathology-specific conferences, main barriers faced by pathologists remain similar to the other groups internationally.

The findings reported in this study mostly reflect the pre-pandemic behavior of author, while during the pandemic most of the conferences were conducted virtually, increasing the number of abstracts submitted but the barriers to full-text publication may not change. Different strategies which can be adopted to overcome the barriers to full text manuscript publication reported by the abstracts’ presenters are:

1. ***PG trainee or junior faculty development as a researcher***: workshops related to research should be planned and executed. CPD activities like courses and workshops can be conducted on ‘How should a researcher manage time?’, ‘How to write a manuscript?’, and ‘How and where to publish?’. Shoko et al. reported that researchers in the developing or LMICs often have inadequate writing skills and workshops can improve these skills [15].
2. ***Mentoring programs***: mentoring programs should be developed by pairing a junior with a senior researcher who can provide guidance in project planning, execution and manuscript writing. As Jatin et al. reported that mentors play a significant role of in guiding, encouraging, and supporting novice researchers [16].
3. ***Cultural change***: small group research meetings, initiatives for formulation of specific task force and working groups on theme-based topics should be developed. Avenues for indexed journals with impact factors should be identified, dissemination of information regarding different grants opportunities to pay for processing/publishing fee for the junior researchers or identify avenues for fee waive off for institutions of LMICs. Keen et al. explores the changes an institutes can bring to support and facilitate researchers to develop a positive culture towards publication, identified that providing editing services and collaborative scientific writing courses can improve the publications [17].
4. ***Resources, awards and appreciations***: one of the barrier to full-text publication was lack of funding/grant to cover for the article processing or publication charges. This can be overcome by giving awards, small grants for publication, and to further motivate young researchers’ societies can give free registration to attend conference etc., hence promoting research culture in a society. The societies and institutes can provide different resources for researchers, like end note for reference citations, software for anlysis, diagrams, plagiarism checking, and language editing services and workshops can be conducted on how to select a journal best suited for the manuscript. A study by Clapton J reported that to enhance research and publication culture the key motivators are sharing of ideas through research meetings, professional development through workshops or courses, giving protected time to researchers to write manuscripts, mentoring, peer recognition and encouragement [18].

The study findings presented here may be particularly useful for researchers from LMICs, however results cannot be extrapolated to the whole country, as these conclusions are mainly based on only 27 questionnaires and larger sample size studies are required in this area.

### Conclusion

The barriers to full manuscript publication were poor time management by the young researcherst and limited writing skills. These findings call for better research training of PG trainees are better equipped to translate their research findings into successful publications and increase the publication rates from the LMICs.

## Limitations

The limitation of the current study was that the reliability testing of questionnaire was not done and not all participants attempted the survey questionnaire and only free papers presented by PG trainees before 2018 were selected.

## Supplementary Information

**Additional file 1: Figure 1.** Proforma of the Survey to assess the publication outcomes of the abstracts presented at the Annual PSCP conferences.

**Additional file 2: Figure 2. a** Abstracts Presented Orally and Published as Full Manuscript. **b **Abstracts Presented as Posters and Published as Full Manuscript.

## Data Availability

Corresponding author have the complete data and it can be shared on appropriate request.

## Abbreviations

PG: Postgraduate
LMIC: Low-middle income country
PSCP: Pakistan Society of Chemical Pathologist
FGD: Focus group discussion
