# Supplementary material for: Publication dynamics: what can be done to eliminate barriers to publishing full manuscripts by the postgraduate trainees of a low-middle income country?
Source: BMC Res Notes. 2022 Jul 15;15:249. doi: 10.1186/s13104-022-06138-5 (PMC9284783; doi:10.1186/s13104-022-06138-5)
Supplement: Supplementary file 1 — Additional file 1: Figure 1. Proforma of the Survey to assess the publication outcomes of the abstracts presented at the Annual PSCP conferences. [file 13104_2022_6138_MOESM1_ESM.docx]

**‘Publication outcomes of the abstracts presented at the 2012-2018 joint Annual conferences of Pakistan Society of Chemical Pathologist’**

Abstract Title: ____________________________

Presented at: ________________________________________

Published: Yes / No,

**If Yes, Citation:** ____________________________________________________________

**If No. kindly encircle one or more reasons of not publishing a conference paper.**

1. Lack of time was the main barrier to publication
2. Pursuit of publication is low priority
3. Difficulty in writing the Discussion/ Literature search
4. Limited writing skills, limited submission skills, and difficulty in starting writing
5. Unable to meet the journal requirements; methodological limitations
6. Negative results or of no/low statistical significance results
7. Lack of funding, in particular for publication fees.
8. Other similar findings studies already published
9. Difficulty with co-authors.
10. Others, Specify ___________________________________________
