# Supplementary material for: Publication dynamics: what can be done to eliminate barriers to publishing full manuscripts by the postgraduate trainees of a low-middle income country?
Source: BMC Res Notes. 2022 Jul 15;15:249. doi: 10.1186/s13104-022-06138-5 (PMC9284783; doi:10.1186/s13104-022-06138-5)
Supplement: Supplementary file 2 — Additional file 2: Figure 2. a Abstracts Presented Orally and Published as Full Manuscript. b Abstracts Presented as Posters and Published as Full Manuscript. [file 13104_2022_6138_MOESM2_ESM.docx]

**Supplemental Figure 1 (a): Abstracts Presented Orally and Published as Full Manuscript**

**Supplemental Figure 1 (b): Abstracts Presented as Posters and Published as Full Manuscript**
